# Tixagevimab/Cilgavimab: Still a Valid Prophylaxis against COVID-19 New Variants?

**DOI:** 10.3390/v16030354

**Published:** 2024-02-25

**Authors:** Anna Gidari, Samuele Sabbatini, Sabrina Bastianelli, Sara Pierucci, Chiara Busti, Elisabetta Svizzeretto, Andrea Tommasi, Carlo Pallotto, Elisabetta Schiaroli, Daniela Francisci

**Affiliations:** 1 Department of Medicine and Surgery, Clinic of Infectious Diseases, “Santa Maria della Misericordia” Hospital, University of Perugia, 06132 Perugia, Italy; sabrina.bastianelli@unipg.it (S.B.); sara.pierucci@unipg.it (S.P.); chiarabusti93@gmail.com (C.B.); elisabetta.svizzeretto@studenti.unipg.it (E.S.); andrea.tommasi@studenti.unipg.it (A.T.); carlo.pallotto@ospedale.perugia.it (C.P.); elisabetta.schiaroli@unipg.it (E.S.); daniela.francisci@unipg.it (D.F.); 2 Department of Medicine and Surgery, Medical Microbiology Section, University of Perugia, 06123 Perugia, Italy; samuele.sabbatini@unipg.it

**Keywords:** COVID-19, SARS-CoV-2, tixagevimab, cilgavimab, variant, immunocompromised, Omicron

## Abstract

Background: this study aims to evaluate the efficacy of tixagevimab/cilgavimab (Evusheld™) against various SARS-CoV-2 variants, including newer Omicron sublineages, in an immunocompromised cohort and in vitro. Study design: Conducted in Italy, this research involves immunocompromised patients who received Evusheld. It evaluates serum neutralization activity against different SARS-CoV-2 strains (20A.EU1, BA.5, BQ.1, XBB.1.5, XBB.1.16, and EG.5) before (T0), after 14 (T1), and after 30 (T2) days from the tixagevimab/cilgavimab injection. Furthermore, the in vitro activity of Evusheld against SARS-CoV-2 VOCs was evaluated. Results: The cohort was composed of 72 immunocompromised patients. The serum neutralizing activity of tixagevimab/cilgavimab-treated patients was notably lower against newer variants such as BQ.1, XBB.1.5, XBB.1.16, and EG.5. Then, the in vitro study detailed specific EC50 values to quantify the activity of tixagevimab/cilgavimab against various SARS-CoV-2 VOCs. Newer variants like BQ.1 and XBB.1.5 exhibited notably lower neutralization, underscoring the challenges in effectively countering the evolving virus. Interestingly, tixagevimab/cilgavimab maintained reduced but still valid activity against EG.5 with an EC50 of 189 ng/mL and Cmax/EC90 of 110.7. Conclusions: Tixagevimab/cilgavimab efficacy wanes against novel subvariants. This underscores the critical need for ongoing adaptation and vigilance in prophylactic strategies to effectively counter the dynamic and unpredictable nature of the COVID-19 pandemic.

## 1. Background

Coronavirus Disease 2019 (COVID-19) has now become endemic with a continuous need for surveillance of its impact on the healthcare system and phases of waning immunity and new exposures being shown. New variants of Severe Acute Respiratory Syndrome Coronavirus 2 (SARS-CoV-2) are continually emerging, with the most widespread variants belonging to the Omicron sublineages [1]. Frail and immunocompromised patients remain the population most at risk for severe disease.

Partial results from our study on the serum neutralization activity of frail patients injected with tixagevimab/cilgavimab (Evusheld™, AstraZeneca, AB, Södertälje, Sweden) against two different SARS-CoV-2 strains were published in September 2023 [2]. The study showed the efficient post-injection serum neutralizing activity (NT90-Abs titer) against the variants 20A.EU1 and Omicron BA.5 in fully vaccinated immunocompromised patients. In particular, before the injection, 61.5% had undetectable neutralizing antibodies (NT90-Abs titer) against BA.5. For both the strains tested (20A.EU1 and BA.5), the titer significantly increased after tixagevimab/cilgavimab injection without significant differences between days 14 and 30. However, the median NT90-Abs titer was 64-fold lower against BA.5 than those against 20A.EU1. In the subsequent three months, only 4/54 patients had COVID-19, but no one required hospitalization.

To the best of our knowledge, few data are available on the most recent and predominant VOCs. In light of this, it is necessary to demonstrate if tixagevimab/cilgavimab maintains activity against the most widespread VOCs and consequently could be still used as prophylaxis for frail patients.

This study aims to establish the post-injection serum NT90-Abs titer and the in vitro activity of tixagevimab/cilgavimab against the most recent VOCs. Here, we present the complete results of the study mentioned above.

## 2. Material and Methods

### 2.1. Design, Setting, and Participants

Immunocompromised patients injected with tixagevimab/cilgavimab as COVID-19 prophylaxis at Santa Maria della Misericordia Hospital, Perugia, Italy, were enrolled from July 2022 to September 2023. Demographic and clinical characteristics, underlying diseases, vaccination history, and previous SARS-CoV-2 infection were obtained from electronic medical records.

Serum samples were withdrawn before (T0), after 14 (T1), and after 30 (T2) days from the tixagevimab/cilgavimab injection.

This cohort was enrolled for a prospective observational study that was approved by the Ethics Committee of the Umbria Region (protocol number 3917/21), and it was conducted following the Declaration of Helsinki. Informed consent was obtained from all subjects involved in the study.

### 2.2. SARS-CoV-2 Strains and Vero E6 Cell Cultures

Our study utilized SARS-CoV-2 strains obtained from patients at Santa Maria della Misericordia Hospital in Perugia, Italy, cultivated in our Biosafety Level 3 (BSL3) virology laboratory. We initiated the experiments by cultivating Vero E6 cells in a T25 flask with Eagle’s Minimum Essential Medium (MEM) enhanced with 10% fetal bovine serum (FBS) and 1% penicillin–streptomycin, maintaining the environment at 37 °C and 5% CO_2_ for 24 h. The subsequent step involved diluting the nasopharyngeal swab transport medium 1:1 with MEM that included 1% penicillin–streptomycin, aiming to mitigate bacterial contamination through a 1-h reaction at 4 °C. This mixture was then inoculated on a monolayer of Vero E6 cells and incubated under the same conditions for 2 h. Following incubation, we replaced the medium with MEM containing 1% FBS and penicillin–streptomycin, continuing the incubation under identical conditions [3,4]. The viral supernatant titer was quantified using the Median Tissue Culture Infectious Dose (TCID50) method [5], and the prepared viral stocks were preserved at −80 °C for future research.

Strains of SARS-CoV-2 20A.EU1 (cluster of lineage B.1.177 and its sublineages circulating in Italy in spring 2020), BA.5, BQ.1, XBB.1.5, XBB.1.16, and EG.5 were used and identified as previously described [6].

### 2.3. SARS-CoV-2 Neutralization Test

The NT90-Abs titers against SARS-CoV-2 were evaluated as previously described [2,7]. We assessed the titers of neutralizing antibodies (NT90-Abs) against SARS-CoV-2 employing 96-well flat-bottom tissue culture plates (Corning Incorporated, New York, NY, USA), adhering to a previously established method [7]. Vero E6 cells (2.5 × 10^4^ per well) were pre-cultured in MEM supplemented with 10% FBS in these plates and incubated at 37 °C and 5% CO_2_ a day before conducting the tests. Serum samples were first heat-inactivated at 56 °C for 30 min and then subjected to two-fold serial dilutions ranging from 1:10 to 1:2560 in MEM containing 2% FBS. These dilutions were placed in 96-well plates in duplicates and combined with a medium containing 50 TCID50 of selected SARS-CoV-2 strains, followed by a 30-min incubation at 37 °C. To ensure accuracy, sera known for their neutralization capacity and plain medium served as positive and negative controls, respectively. These mixtures were then added to the plates with Vero E6 cells and incubated for 72 h under the same temperature and CO_2_ conditions. After this period, the medium was discarded, and the cells were fixed and stained using a solution of 0.25% crystal violet and 10% formalin for 30 min at room temperature. After washing off excess stain, absorbance at 595 nm was measured using a microtiter plate reader (Multiskan Fc Photometer, Thermo Fisher Scientific, Waltham, MA, USA). The neutralizing antibody titer was identified as the highest dilution, achieving a ≥90% reduction in cytopathic effect (CPE) compared to the virus control.

### 2.4. EC50 Determination

An in vitro study was performed to assess tixagevimab/cilgavimab half-maximal effective concentration (EC50) on SARS-CoV-2 VOCs as previously described, with some modifications [8]. Briefly, Vero E6 cells were seeded overnight in 96-well plates, and six concentrations of tixagevimab/cilgavimab were prepared by 1:4 dilution (5000–4.9 ng/mL). The antibodies (both 50 μL/well) and SARS-CoV-2 strains (100 μL, 0.002 MOI) were added in triplicate to the cells. Plates were incubated 48 h at 37 °C with CO_2_ 5% prior to viral quantification by plaque reduction assay [9]. Four-parameter variable slope regression modeling of tixagevimab/cilgavimab dose-response was performed.

The maximum achievable plasma concentrations at an approved dose in humans (Cmax) for each antibody was deduced from literature data and used for Cmax/(EC50 or EC90) ratio. Values above 1 are assumed to be a good indicator of potential human efficacy [10].

### 2.5. Statistical Analysis

GraphPad 8.3 software (GraphPad Software, San Diego, CA, USA) was used for all statistical analysis. Data were tested for normality using the Kolmogorov-Smirnov test. For descriptive analyses, data were shown as mean with the respective standard deviation (SD), median with interquartile range (IQR), or percentage as appropriate.

Data following a normal distribution were examined using one-way repeated measures ANOVA and Bonferroni’s multiple comparison test. For variables not meeting parametric criteria, analyses were conducted using the Friedman test and Dunn’s multiple comparison test as appropriate. A *p*-value < 0.05 was considered significant.

## 3. Results

The cohort was composed of 72 immunocompromised patients injected with tixagevimab/cilgavimab.

Demographic and clinical characteristics are summarized in Table 1. The mean age was 64.6 ± 12.0 years, and 37 (51.4%) were male. The majority of participants (51/72, 70.8%) were diagnosed with onco-hematological malignancies. Additionally, a significant portion (69/72, 95.8%) had completed their vaccination regimen. On average, the interval between receiving the last vaccine dose and the administration of the tixagevimab/cilgavimab injection was approximately 229.9 days (SD 94.7 days). All subjects were tested for SARS-CoV-2 NT90-Abs before the injection (T0), and a low median titer was found against 20A.EU1 strain (20, interquartile range, IQR, 0–40) while it was <10 against the other strains.

About a third of patients (24/72, 33.3%) showed a NT90-Abs titer <10 against all strains, including 20A.EU1. No differences in the NT90-Abs titer were observed between patients with previous SARS-CoV-2 infection and those without, across all time points. Similarly, receiving three or four doses of the SARS-CoV-2 vaccine did not make any difference.

**Figure 1:**
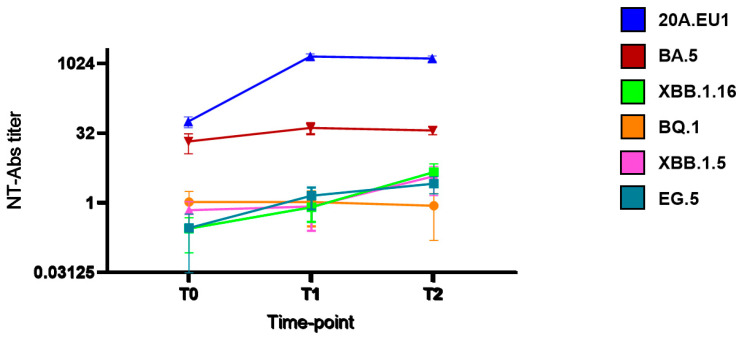
Serum neutralization activity after tixagevimab/cilgavimab injection: neutralizing antibodies (NT90-Abs) titer decay curve for different SARS-CoV-2 variants. Time points: before the injection (T0), after 14 days (T1), and after 30 days (T2).

As shown in Figure 1, after tixagevimab/cilgavimab injection, we observed a significant increase in NT90-Abs reaching the peak after 14 days (T1) against 20A.EU1 (640, IQR 320–640, p) and BA.5 (20, IQR 10–20). The titer was roughly maintained after 30 days (T2). However, the NT90-Abs titer remained <10 against BQ.1, XBB.1.5, XBB.1.16, and EG.5 VOCs without differences compared to the pre-injection value.

**Figure 2:**
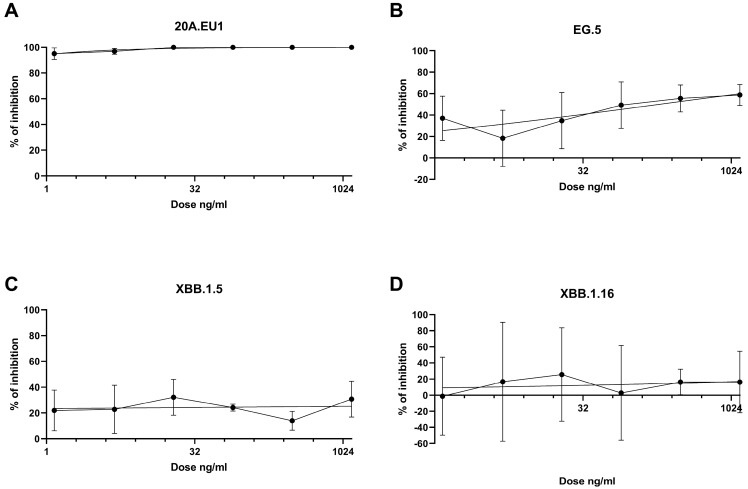
Dose-response inhibition test of tixagevimab/cilgavimab (1.2-1250 ng/mL) against 20A.EU1 (**A**), EG.5 (**B**), XBB.1.5 (**C**), and XBB.1.16 (**D**) strains of SARS-CoV-2 in Vero E6 cells (MOI 0.002). After 48 h of incubation on 96-well plates, supernatant titers were determined with plaque assay. Effective concentrations (EC50 and EC90) were calculated with four-parameter variable slope regression modeling. The data are presented as % of inhibition.

The antibodies were then tested in a yield reduction assay to subsequently determine the viral titer on cell supernatants. Four-parameter variable slope regression modeling of tixagevimab/cilgavimab dose-response showed EC50 of 0.01 ng/mL (95% confidence interval, CI, 0.00002-0.18 ng/mL) and EC90 of 0.51 ng/mL (slope of 0.64; CI 0.30-1.51) for the 20A.EU1 SARS-CoV-2 strain (Figure 2A). On the EG.5 strain, the antibodies showed EC50 of 189.7 ng/mL (95% CI, 36.7–16,155) and EC90 of 6.1 × 10^6^ ng/mL (slope of 0.21; 95% CI 0.06-0.39) (Figure 2B). Against XBB.1.5 and XBB.1.16, the antibodies did not show significant activity; consequently, it was not possible to determine EC50 (Figure 2C,D).

The achievable maximum plasma concentrations for tixagevimab and cilgavimab are 21.1 µg/mL (150 mg intramuscular, IM) and 21.3 µg/mL (150 mg IM), respectively [11]. For both of the antibodies, we approximate the Cmax to be 21 µg/mL. According to our data, Cmax/EC50 and Cmax/EC90 against 20A.EU1 were 2.1 × 10^6^ and 41.2 × 10^3^. Regarding the EG.5 strain, Cmax/EC50 was 110.7 and Cmax/EC90 was 0.003.

## 4. Discussion

The combination of the two long-acting antibodies tixagevimab/cilgavimab was approved in the European Union (EU) at the beginning of 2022 for pre-exposure prophylaxis of COVID-19, showing reduced risk of developing symptomatic COVID-19 and 6 months of lasting protection [12]. Later, Evusheld was then approved in the EU for treatment of COVID-19 [13].

Evusheld was demonstrated to retain potent neutralizing activity (fold-change IC50 < 3.0) against the SARS-CoV-2 VOCs Alpha, Beta, Gamma, and Delta compared to the USA-WA/1/2020 and AUS/IC01/2020 reference strains [14]. However, the emergence of Omicron variants has challenged the validity of this prophylaxis. In the absence of other effective prophylaxis against SARS-CoV-2 in immunocompromised patients, does it still make sense to use Evusheld?

Several studies have reported clinical data about different frail populations such as hematological malignancies, kidney and liver transplant recipients, patients with end-stage renal disease on chronic hemodialysis, autoimmune diseases, and other important comorbidities. Most of these studies observed that in populations unresponsive to vaccination, tixagevimab/cilgavimab pre-exposure prophylaxis was associated with lower incidence of COVID-19 and severe infection [15,16,17,18,19]. These clinical studies were conducted across various timeframes, resulting in patient exposure to diverse variants of concern (VOCs). Despite these differences, the therapeutic benefits were consistently observed.

A study suggested that individuals with B-cell malignancies, even after receiving tixagevimab/cilgavimab as pre-exposure prophylaxis alongside therapies that deplete B-cells or have undergone hematopoietic stem cell transplantation within 3 to 6 months, remain susceptible to breakthrough COVID-19. This vulnerability is likely due to altered immune responses, particularly in B and T lymphocytes, characteristic of B-cell malignancy patients. The connection between when tixagevimab/cilgavimab is administered and the onset of infection seems negligible. Importantly, the incidence of hospitalization among those who contracted the virus was low, with no fatalities reported, indicating that tixagevimab/cilgavimab prophylaxis might play a crucial role in mitigating the severity and preventing death in cases of breakthrough infections among these patients [20]. Despite these results, it is difficult to establish the real impact of the new VOCs on Evusheld efficacy. Furthermore, monoclonal antibody therapy that is not fully protective may cause induction of new mutations, which are associated with prolonged infection but no clinical worsening. From that, some substitutions have been associated with tixagevimab/cilgavimab in different SARS-CoV-2 lineages, including BQ.1 and XBB [21].

According to our results, the most recent Omicron sublineage VOCs demonstrated a greater escape from serum neutralization than BA.5. In particular, the in vitro serum NT90-Abs was undetectable for BQ.1, XBB.1.5, XBB.1.16, and EG.5 strains. Similarly, Zhao et al. observed that XBB.1.5 showed the greatest escape activity among the subvariants [22], and the experiments with pseudoviruses harboring SARS-CoV-2 spike proteins of BA.5, XBB.1.5, and XBB.1.16 produced similar results [23]. Other studies demonstrated that Evusheld has lost antiviral efficacy against XBB subvariants and BQ.1.1 [24,25,26]. To the best of our knowledge, no data are available in the literature about the serum neutralization or in vitro activity of Evusheld against the EG.5 strain. Only Zhang et al. performed a neutralization assay against EG.5.1 pseudotyped particles as a surrogated model of SARS-CoV-2 cell entry and neutralization. The authors found that tixagevimab/cilgavimab was not able to inhibit cell entry [27]. Interestingly, despite our study finding low serum neutralization activity, unlike the other strains, the SARS-CoV-2 EG.5 strain was demonstrated to be inhibited by tixagevimab/cilgavimab, less efficiently than the 20A.EU1 strain, but at concentrations reachable in vivo, as evidenced by the Cmax/EC50 being largely above 1.

The limits of this study are the small cohort, the use of an in vitro model that could affect the antibodies efficacy, the lack of other less common variants, and the use of non-human cell lines.

In conclusion, it is not clear if, in the absence of other efficient prophylaxes, Evusheld could be an option for immunocompromised patients that do not respond to vaccines. Despite its efficiency being highly afflicted by the emergence of Omicron variants, tixagevimab/cilgavimab still maintained activity against some variants, such as EG.5, that had a global prevalence around 30% at the end of 2023 [28]. Furthermore, our clinical data almost univocally reported a benefit of this prophylaxis in terms of infection and severity of the disease. We will certainly have to wait for the release of new monoclonal antibodies for a complete effectiveness of this type of treatment, but a positive effect of tixagevimab/cilgavimab in fragile and high-risk patients is still conceivable.

## Figures and Tables

**Table 1:** Demographic characteristics of the cohort.

Characteristics	N 72
Age, mean (SD) [range], years	64.6 (12.0) [34.8-89.3]
Sex, male N (%)	37 (51.4)
Comorbidities:	
Onco-hematological malignancy, N (%)	51 (70.8)
Primary immunodeficiency, N (%)	5 (6.9)
Kidney transplantation, N (%)	4 (5.5)
Chronic kidney disease, N (%)	3 (4.1)
Secondary immunodeficiency, N (%)	1 (1.1)
Autoimmune disease in immunosuppressant therapy	8 (11.1)
Vaccination, N (%)	69 (95.8)
3 doses	52 (75.3)
4 doses	18 (25.0)
5 doses	1 (1.4)
Time from last vaccine dose to monoclonal injection, days, mean (SD)	229.9 (94.7)
Previous SARS-CoV-2 infection, N (%)	24 (33.3)

Footnotes: SD, standard deviation; N, number of patients.

## Data Availability

The data presented in this study are available on request from the corresponding author.
